# Dynamic Evolution of Rht-1 Homologous Regions in Grass Genomes

**DOI:** 10.1371/journal.pone.0075544

**Published:** 2013-09-24

**Authors:** Jing Wu, Xiuying Kong, Chao Shi, Yongqiang Gu, Cuiyun Jin, Lizhi Gao, Jizeng Jia

**Affiliations:** 1 Key Laboratory of Crop Germplasm Resources and Utilization, Ministry of Agriculture, the National Key Facility for Crop Gene Resources and Genetic Improvement, Institute of Crop Science, the Chinese Academy of Agricultural Sciences, Beijing, China; 2 Plant Germplasm and Genomics Center, Germplasm Bank of Wild Species in Southwest China, Kunming Institute of Botany, the Chinese Academy of Sciences, Kunming, China; 3 United States Department of Agriculture, Agricultural Research Service, Western Regional Research Center, Albany, California, United States of America; Ben-Gurion University, Israel

## Abstract

Hexaploid bread wheat contains A, B, and D three subgenomes with its well-characterized ancestral genomes existed at diploid and tetraploid levels, making the wheat act as a good model species for studying evolutionary genomic dynamics. Here, we performed intra- and inter-species comparative analyses of wheat and related grass genomes to examine the dynamics of homologous regions surrounding *Rht*-*1*, a well-known “green revolution” gene. Our results showed that the divergence of the two A genomes in the *Rht-1* region from the diploid and tetraploid species is greater than that from the tetraploid and hexaploid wheat. The divergence of D genome between diploid and hexaploid is lower than those of A genome, suggesting that D genome diverged latter than others. The divergence among the A, B and D subgenomes was larger than that among different ploidy levels for each subgenome which mainly resulted from genomic structural variation of insertions and, perhaps deletions, of the repetitive sequences. Meanwhile, the repetitive sequences caused genome expansion further after the divergence of the three subgenomes. However, several conserved non-coding sequences were identified to be shared among the three subgenomes of wheat, suggesting that they may have played an important role to maintain the homolog of three subgenomes. This is a pilot study on evolutionary dynamics across the wheat ploids, subgenomes and differently related grasses. Our results gained new insights into evolutionary dynamics of *Rht-1* region at sequence level as well as the evolution of wheat during the plolyploidization process.

## Introduction

"Evolutionary dynamics" can be used to study evolutionary mechanisms and processes. Because of the presence of homoeologous genes, allopolyploids are suitable for studying sequence structure, nucleotide diversity, and evolutionary relationships at homoeologous loci, providing insights into the evolutionary dynamics of functionally important loci [1,2]. Patterns and mechanisms of evolutionary dynamics underlying polyploid evolution, which are still poorly understood, can have an impact on breeding programs particularly for genetic improvement of new crop species such as *Triticale* .

Bread wheat *Triticum aestivum* L., which represents one of the best-characterized examples of genome polyploidization, was evolved through the process of two spontaneous hybridization events. The first one occurred some 500,000 years ago between a diploid species *T* *. urartu* (AA) and an unknown B genome species probably belonging to the *Sitopsis* group of *Ae* *. speltoides* , giving rise to a tetraploid AABB genome species. The second hybridization event took place some 8,000-10,000 years ago between the tetraploid AABB genome species and a diploid *Ae* *. tauschii* , the D genome donor, giving rise to current-day bread wheat (2n=6x=42, AABBDD) [3]. Compared with other allopolyploids, wheat is considered to be a young polyploid as a result of relatively recent speciation. Wheat thus has been long employed as a classical system for studying the process of allopolyploidization in flowering plants.

Comparative genomics is often used to investigate evolutionary relationships of genomes from different species and serves as an efficient tool for studying genome sequence composition, structure, gene duplications, origin of new genes and colinearity between different genomes [4-8]. Recently, bread wheat has been used to study the origin of species, chromosome rearrangements, structural variations, and amplification of transposable elements in the polyploidization process [7-10]. The genome of hexaploid wheat is about 16,000 Mb, and contains up to 80% of repetitive sequences [11]. Furthermore, the complexity of the bread wheat genome as an allopolyploid makes it fairly challenging to be completely sequenced. To gain the first view of the wheat genome, several studies have focused on comparative studies on important genes and flanking genomic regions in wheat, including D-hordein, HMW-glutenin, Acc, Hardness, and Q gene loci [4,5,9,12,13]. These studies have obtained in-depth knowledge of the composition and organization of genomes and revealed subtle forms of conservation and divergence between homologous genomic regions. To date, the majority of comparative sequence analyses have centered on either wheat polyploids and their diploid ancestors or grass genomes of distantly related diploid species. Hence, evolutionary dynamics of loci controlling important traits after recent polyploidization in wheat species in comparison with other related grasses representing broad lineages has not been adequately addressed. A detailed sequence comparison of the genomes in wheat polyploids, their diploid ancestors and related grasses will allow for a better understanding of the mechanisms determining these evolutionary events during polyploidization.

Wheat plant reduced height-1 (*Rht-1*) genes play a major role in modern agriculture. The *Rht-B1b* and *Rht-D1b* alleles of the *Rht-B1* and *Rht-D1* genes of wheat have been widely used since the start of the green revolution, being an important component for the improvement of crop yield. Some alleles of *Rht-1* conferring dwarfism have been cloned [14-17]. However, little is known about the molecular basis of the evolutionary events that have shaped the *Rht-1* locus regions in wheat. The availability of several sequenced grass genomes, representing diverse lineages, for example, *Oryza sativa*, *Sorghum* *bicolor* , *Brachypodium* *distachyon* , *Zea mays* and *Setaria* *italic* [18-24], provides an unprecedented opportunity to better understand genomic composition and evolution of *Rht-1* homologous genomic regions across different grass species.

In this study, we identified and sequenced *Rht-1* homoeologous BACs from the wheat diploid, tetraploid and hexaploid genomes of *T* *. urartu* , *Ae* *. tauschii* , *T* *. durum* and *T. aestivum*. We investigated the molecular basis of genomic rearrangements that occurred at the *Rht-1* locus by comparing corresponding sequences of diploid, tetraploid, and hexaploid wheat species ( *Triticum* and *Aegilops* ), which diverged relatively recently. We also focused on the characterization of sequence variation to investigate molecular evolution of the wheat *Rht-1* homologous genomic regions during the process of polyploidization. To gain a broad insight into the patterns and evolutionary mechanisms of the *Rht-1* homologous regions along diverse grass lineages, we also included and compared with the orthologous regions of *O. sativa*, *B* *. distachyon* , *S.* bicolor, *Z. mays* and *S* *. italica* . The comparative analyses of these orthologous regions provided the first view of sequence divergence on a large scale in the wheat A, B, and D genomes, and enhanced our understanding of molecular evolution of *Rht-1* genomic regions across diverse lineages of grasses.

## Materials and Methods

### Screening and sequencing of the wheat BACs

The diploid and tetraploid wheat BAC clones of the A and B genomes were selected from the *T* *. urartu* and *T* *. durum* (cv. Langdon) BAC libraries by screening with Southern hybridization. The hexaploid wheat BAC clones of A, B and D subgenomes were obtained from the *T. aestivum* (cv. Chinese spring) BAC library by screening with PCR primers specific to the *Rht-B1b* and *Rht-D1b* genes. The diploid wheat BAC clone of D genome was selected from the *Ae* *. tauschii* (AL8/78) with the same PCR primers. A total of four wheat BAC libraries were used to isolate the BACs covering the *Rht-D1b* or homologous genes. The diploid libraries were first screened by using *T* *. urartu* and *Ae* *. tauschii* (AL78/8) with a coverage of 1.8-fold and 2-fold, respectively. Then the tetraploid library was constructed with coverage of 5.1-fold using *T* *. durum* (cv. Langdon), which was kindly provided by Dr. Yong-Qiang Gu. Finally, the hexaploid library was constructed from Aibai/CS near-isogenic line (NIL) of *T. aestivum* and the coverage was estimated to be 6.5-fold.

*E. coli*-freed DNAs from BAC clones were isolated with the QIAGEN Large-Construct Kit, mechanically sheared into fragments of 2-5Kb by Hydroshear (Gene Machines). The 2-5Kb fragments were blunt-ended with mung bean nuclease and dephosphorylated with Shrimp Alkaline Phosphatase (SAP). Then they were ligated into a pCR4-TOPO vector and transformed into TOP10 electro-competent cells. Individual clones were sequenced from both forward and reverse directions using ABI BigDye3.1 terminator chemistry and analyzed on an ABI 3730XL automated capillary sequencer. Preassembly and assembly analyses of the sequencing reads were performed by using PHRED [25], and assembled through the Lasergene v7.10 software (http://www.dnastar.com/) with the parameters Match Size 40 and Minimum Match Percentage 98. Gaps were closed and weak consensus regions strengthened by either direct sequencing of subclones using primer walking with adding dGTP mix and DMSO in the sequencing reaction system. VISTA family tool (http://genome.lbl.gov/vista/index.shtml) was used to identify the conserved non-coding sequences (CNSs) in the *Rht-1* region [26].

### Sequence assembly and annotation

For the annotation of BAC sequences, all known repetitive elements were first identified through BLAST searches against the database for Triticeae repetitive elements (TREP) (http://wheat.pw.usda.gov/ITMI/Repeats/), TIGR Plant Repeat Databases (http://plantrepeats.plantbiology.msu.edu/index.html) and RepeatMasker (http://www.repeatmasker.org/cgi-bin/WEBRepeatMasker). Their annotation was next performed with minor modifications (http://wheat.pw.usda.gov/ITMI/Repeats/ gene_annotation.pdf). Here, gene predictions were performed mainly by using the program FGENESH with training sets of the monocots including maize, rice, wheat and barley (http://www.softberry.com). In addition, GENSCAN was complementarily run against the obtained wheat BAC sequences with the database of maize (http://genes.mit.edu/GENSCAN.html). MicroRNAs (miRNAs) were detected against the miRBase Database (Release 18.0) (http://www.mirbase.org/) [27]. Then these candidate miRNA sequences were folded to test their secondary structures using M-fold web [28]. Target predictions were performed to search the wheat EST (http://www.tigr.org/tdb/e2k1/tae1/index.shtml) and KOMUGI databases (http://www.shigen.nig.ac.jp/wheat/komugi/) for miRNA complementary sequences, allowing up to three mismatches and with no gaps between miRNAs and target mRNAs [29]. The software Gepard-1.2 (http://www.warezkeeper.com/ gepard-v.1.2-crack-serial-keygen-download.html) was used for the dot-plot analysis, in which sequence criteria of 60% was taken with a window size of 40bp. In addition, *Rht-1* homologous genomic regions were identified and downloaded from the genomes of *Setaria* *italic* (http://www.phytozome.net/foxtailmillet.php), *Brachypodium* *distachyon* (http://www.brachypodium.org/), *Oryza sativa* (http://rice.plantbiology.msu.edu/), *Sorghum* *bicolor* (http://www.phytozome.net/sorghum) and *Zea mays* (http://www.plantgdb.org/ZmGDB/). For the purpose of reasonably comparative analyses, these sequences were annotated through the same standard as used in wheat sequences.

### Data analyses

Full-length elements were aged by comparing their 5’ and 3’ LTR sequences [30]. The composition distances of the two LTR sequences were calculated by MEGA 4.0 using the Kimura-2 model to estimate the insertion times of LTR-retrotransposons [31]. In this study, we used the average substitution rate of 6.5×10^-9^ substitutions per synonymous site per year, estimated from the *adh1* and *adh2* loci of grasses [32]. The time (*T*) since element insertion was calculated by using the formula *T*=*K*/2*r*, where *T* is the time of divergence, *K* is the divergence, and *r* is the substitution rate [33]. The molecular clock was calibrated using 60 MYA for divergence of *T. aestivum* from *Z. mays*. MEGA5.0 was also used to generate neighbor-joining (NJ) trees with bootstrap values. codeml module of PAML version 4.7 with the F3X4 codon frequency model was used to calculate the pairwise nonsynonymous substitutions rates (*Ka*) and synonymous substitutions rates (*Ks*). While, the nucleotide substitutions of UTRs and introns were calculated by baseml module [34].

## Results

### Sequencing and analysis of the wheat BACs

A total of seven BAC clones containing the *Rht-1* homologous regions of *T* *. urartu* , *Ae* *. tauschii* , *T* *. durum* and *T. aestivum* were selected for sequencing. Among them, the three BAC clones from the A genome, two from the B genome, and two from the D genome were screened, respectively (Table 1).

**Table 1:** The assembled BACs from the wheat species and homologous genomic regions of the related grass species.

**Genome**	**BAC No.**	**BAC libraries**	**Sequence length (bp**)	**Gaps**	**Chromosomes**
**A genome**	105A8	*Triticum* *urartu*	100,141	0	4A
	1051O6	*T* *. durum* (Langdon)	122,178	2	4A
	351D1	*T. aestivum* (Chinese spring)	96,804	0	4A
**B genome**	315P18	*T* *. durum* (Langdon)	151,879	0	4B
	17O6	*T. aestivum* (Chinese spring)	108,070	1	4B
**D genome**	C4	*Aegilops* *tauschii*	189,300	1	4D
	1J9	*T. aestivum* (Chinese spring)	207,530	0	4D
** *Brachypodium* **		*Brachypodium* *distachyon* 21	37,675	5	1
**Sorghum**		*Sorghum* *bicolor* (L.) Moench	58,435	3	1
**Rice**		*Oryza sativa* ssp. *indica*	65,936	0	3
**Maize**		*Zea mays* ssp. *mays* L. (B73)	190,275	21	1
**Foxtail millet**		*Setaria* *italica* (Yugu1)	24,234	0	Not Determined

Annotation of the BAC sequences indicated that *Fragile-X-F-like* (gene 1), *DUF6*-like (gene 2) and *Rht*-*1* (gene 3) genes were all supported by the wheat ESTs in the Genbank. These three genes were shared among the A, B, and D genomes (Table S1, Figure S1A, B, C). The RT-PCR analyses revealed that the *Rht-1* homologous genes of the A, B, and D genomes were ubiquitously expressed at all developmental stages and different tissues examined, while the *DUF6*-like gene was only expressed in the stem and seed, indicative of its conditional expression in wheat (Figure S1D). To further examine transcriptional regulation of *Rht-1* homologous genes, we analyzed the 1,500-bp upstream promoter sequences of these *Rht-1* homologous genes using the PlantCARE database. Blast searches found highly conserved essential *cis*-regulatory elements of promoter, including TATA, CAAT, and GC-box across the investigated species; SP1 motif and MBS were also detected in all these subgenomes. G-box (CACGTG) was further found to be shared by the A and B subgenomes but not the D subgenome of *T. aestivum*; 5’ UTR Py-rich stretch element (TTTCTTCTCT), which usually confers high transcription levels, were found in the B and D subgenomes of *T. aestivum* but not in the A subgenome of *T. aestivum*. A gibberellin-responsive element, P-box (CCTTTTG), was only found in the D subgenome of *T. aestivum* (Table S2).

The repetitive sequences were the major components in the sequenced genomic region, consisting of a wide variety of transposable elements (TEs). The repetitive sequences of individual BACs ranged from 37.36% (BAC 105A8) to 75.31% (BAC C4) (Table S3). Among the TEs, the content of DNA transposons ranged from 0.63% to 11.44%, while retrotransposons account for 35.63% to 66.14%. Retrotransposable elements, Copia, Gypsy, CACTA, and MITEs were found to be the most important superfamilies resided within the homologous genomic regions (Table S4, S5 & S6). A total of 13 complete/intact LTR retrotransposons were identified with variable insertion times. Of them, the oldest LTR retrotransposon was RLG_Fatima_315P18-1 and RLG_Fatima_17O6-1 with their insertion times around ~2.15 MYA, whereas RLC_WIS_1051O6-2 and RLC_WIS_351D1-2 were the youngest elements with an insertion time of ~0.27 MYA (Table S4). In addition, four candidate miRNAs (TamiR1122, TamiR1137, TamiR1132 and TamiR1121) and Simple Sequence Repeat (SSR) were identified in these BAC sequences (Tables S7 & S8).

### Genomic divergences of the homologous *Rht-1* gene regions at different ploidy levels

**Figure 1:**
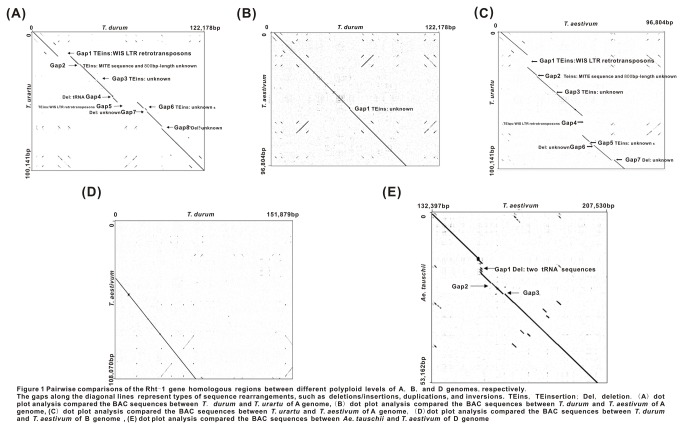
Pairwise comparisons of the *Rht-1* gene homologous regions between different polyploid levels of A, B, and D genomes, respectively. The gaps along the diagonal lines represent types of sequence rearrangements, such as deletions/insertions, duplications, and inversions. TEins, TEinsertion; Del, deletion. (A) dot plot analysis compared the BAC sequences between *T* *. durum* and *T* *. urartu* of A genome, (B) dot plot analysis compared the BAC sequences between *T* *. durum* and *T. aestivum* of A genome, (C) dot plot analysis compared the BAC sequences between *T* *. urartu* and *T. aestivum* of B genome, (D) dot plot analysis compared the BAC sequences between *T* *. durum* and *T. aestivum* of B genome, (E) dot plot analysis compared the BAC sequences between *Ae* *. tauschii* and *T. aestivum* of D genome.

To characterize the sequence variation in the *Rht-1* homologous regions of the wheat genomes, we performed dot matrix analyses between pairs of corresponding genomes from two different ploidy levels. Genomic divergences were designated as gaps in the main matrix diagonal lines (Figure 1). The average conserved fragment size (CFS) and conserved sequence ratio (CSR) was calculated based on sequence alignments to evaluate the sequence divergence (Table 2).

**Table 2:** The average conserved fragment size and conserved ratio between different wheat genomes.

**Genome**	**Homologous RegionsSize (bp**)	**Conserved Fragment Size (bp**)	**Conserved Sequence Ratio (%**)	**Average Conserved Fragment Size (bp**)
**A *T* *. urartu* : A *T* *. durum* **	123,270	82,563	67.0%	9,173
**A *T* *. urartu* : A *T. aestivum***	99,054	71,138	71.8%	10,162
**A: *T* *. urartu* -B: *T* *. durum* **	25,902	12,173	46.9%	1,739
**A *T* *. durum* : A *T. aestivum***	96,932	95,924	98.9%	47,962
**B *T* *. durum* : B *T. aestivum***	68,851	68,767	99.9%	34,383
**B: *T* *. durum* -D: *Ae* *. tauschii* **	63,512	13,569	21.4%	3,392
***DAe.tauchii* : D *T. aestivum***	81,518	76,344	93.7%	19,086
**A *T. aestivum* : B *T. aestivum***	32,422	12,911	39.8%	2,582
**A *T* *. urartu* : D Ae*.tauchii***	63,619	14,427	22.7%	3,606
**A *T. aestivum* : D *T. aestivum***	58,405	16,530	28.3%	2,066
**B *T. aestivum* : D *T. aestivum***	58,321	14,105	24.2%	3,526

Sequence comparisons of the *Rht-1* homologous regions among the three A genomes showed that they shared all the three genes with highly conserved collinearity. These collinear genes have the same transcriptional orientations and exon/intron structures at different ploidy levels (Figure S1A, B, C). However, when both CSR and CFS were used to examine the sequence variation, it is found that the CSR and CFS between tetraploid and hexaploid were much higher than that diploid Vs tetraploid and diploid Vs tetraploid (98.9%,67.0%, 46.9% and 47,962bp, 9,173bp 1,739bp, respectively, Table 2). This is in accordance with more gaps (8 gaps) detected in the overlapped regions between the diploid and tetraploid genomes (Figure 1A). Analysis of these gap regions revealed that LTR retroelements, WIS-type element (WIS-2), WIS-type element (WIS-3) and *Copia*-type retroelement fragment caused the Gap1 and Gap2, a DNA transposon MITE insertion caused Gap2, a *tRNA* element caused gap 4, and unknown sequence deletions caused Gap7 and Gap8. Gap3 and Gap6 were caused by two high GC content regions that were unable to sequence through. In contrast, only one gap was observed between the A genomes from tetraploid and hexaploid species. This gap was generated by an insertion of 800-bp sequence in the *T. aestivum* sequence (Figure 1B, S2A). Because sequences of tetraploid and hexaploid genome have high conservatism, seven gaps were observed between diploid and hexaploid species, similar to those between tetraploid and hexaploid genome (Figure 1C, S2A). Apparently, greater genomic divergences were present between the diploid and tetraploid species as compared to that between the tetraploid and hexaploid wheat.

The CFS and CSR values in the 68,851 bp overlapping region of the B genomes between tetraploid and hexaploid were very similar with the two A genomes from the polyploid wheat (Figure 1D, S2B and Table 2). Only a few sequence differences were identified during the evolutionary process from the tetraploid to hexaploid wheat, including a 43bp insertion of unknown sequence encompassing the duplication of TGCGGGCATGCGGCCGATGGCGG A.

The divergences between diploid and hexaploid (no tetraploid for D genome in wheat) of the D genome were also examined (Figure 1E, S2C and Table 2). A total of 81,518bp overlapping region was observed aligned between the two D genomes from the diploid and hexaploid species. 93.7% of which was conserved, including LTR retrotransposon, CACTA element, MITEs of Stowaway, tRNA sequences of SINEs, SSR sequences of (CT) 23, (GAA) 8 and (CGGT) 5 as well as two predicted genes. Only three gaps were observed in this region; one was caused by tRNA element deletion in *Ae* *. tauschii* , and other two gaps might be caused by sequencing issues due to the GC-rich genomic regions.

The nucleotide substitution rates (NSR) were also employed to analyze the sequence divergence. We estimated NSR between the diploid, tetraploid and hexaploid wheat of A, B, and D genomes, respectively, based on pairwise comparisons of *Rht-1* and *DUF6*-like genes (Table 3; Table S9). Few or no nucleotide substitutions were detected in the two homologous A and in the two homologous B genomes from *T* *. durum* and *T. aestivum* and in the two homologous D genomes from *Ae* *. tauschii* and *T. aestivum*. However, when the diploid A genomes was compared with the A genomes from the polyploid wheat, higher nucleotide divergences were found, suggesting that there were more divergences from diploid to polyploid but less from tetraploid to hexaploid in the A genome, also less from diploid to hexaploid in D genome.

**Table 3:** The estimated substitution rates between the wheat and related grasses based on pairwise comparisons of *Rht-1* gene*.

Pairwise Genomes	5’UTR regions		Coding-regions				3’UTR regions	
	Length (bp)	K	Length (bp)	Ks	Ka	Ka/Ks	Length (bp)	K
**A: *T* *. urartu* -A:T*.durum***	627	0.0067	1860	0.0329	0.0000	0.0010	805	0.0029
**A: *T* *. urartu* -A:T*.aestivum***	627	0.0067	1860	0.0329	0.0000	0.0010	805	0.0029
**A: *T* *. urartu* -B: *T* *. durum* **	627	0.1210	1875	0.3227	0.0045	0.0140	822	0.0886
**A: *T* *. urartu* -D: *Ae* *. tauschii* **	630	0.0482	1876	0.2003	0.0146	0.0729	814	0.0629
**A: *T* *. durum* -A:T*.aestivum***	623	0.0000	1860	0.0000	0.0000	0.0010	791	0.0000
**B: *T* *. durum* -B: *T. aestivum***	579	0.0000	1863	0.0080	0.0000	0.0010	795	0.0000
**B: *T* *. durum* -D: *Ae* *. tauschii* **	629	0.1239	1881	0.2558	0.0159	0.0622	827	0.0600
**D: *Ae* *. tauschii* -D: *T. aestivum***	607	0.0034	1869	0.0000	0.0000	0.0010	784	0.0059
**A: *T. aestivum*-B: *T. aestivum***	639	0.1321	1872	0.2949	0.0045	0.0153	837	0.0925
**A: *T. aestivum*-D: *T. aestivum***	636	0.0513	1872	0.2409	0.0145	0.0602	806	0.0670
**B: *T. aestivum*-D: *T. aestivum***	637	0.1288	1878	0.2487	0.0159	0.0639	830	0.0605
**D: *T. aestivum-B.distachyon***	639	0.4931	1899	0.8594	0.0404	0.0470	796	0.4439
**D: *T. aestivum-S.bicolor***	631	0.5740	1917	0.9806	0.0677	0.0690	845	0.6488
**D: *T. aestivum-S.italica***	655	1.1462	1908	0.8741	0.0551	0.0630	869	0.5472
**D: *T. aestivum-O.sativa***	655	0.7067	1896	1.0089	0.0614	0.0609	799	0.6211
**D: *T. aestivum-Z.mays***	623	0.5713	1917	1.2046	0.0584	0.0485	802	0.6925

* Note that 0.0000 in this table is the value after rounding, not the really zero.

### Pair-wise comparisons of the orthologous *Rht-1* gene regions among the A, B, and D subgenomes

**Figure 2:**
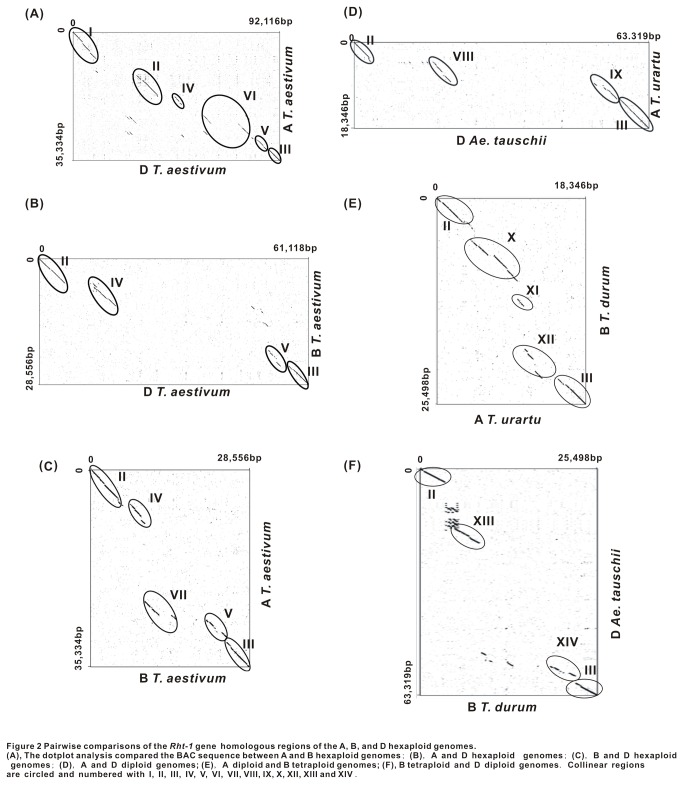
Pairwise comparisons of the *Rht-1* gene homologous regions of the A, B, and D hexaploid genomes. (A) The dotplot analysis compared the BAC sequence between A and B hexaploid genomes; (B), A and D hexaploid genomes; (C), B and D hexaploid genomes; (D), A and D diploid genomes; (E), A diploid and B tetraploid genomes; (F), B tetraploid and D diploid genomes. Collinear regions are circled and numbered with I, II, III, IV, V, VI, VII, VIII, IX, X, XII, XIII and XIV.

**Figure 3:**
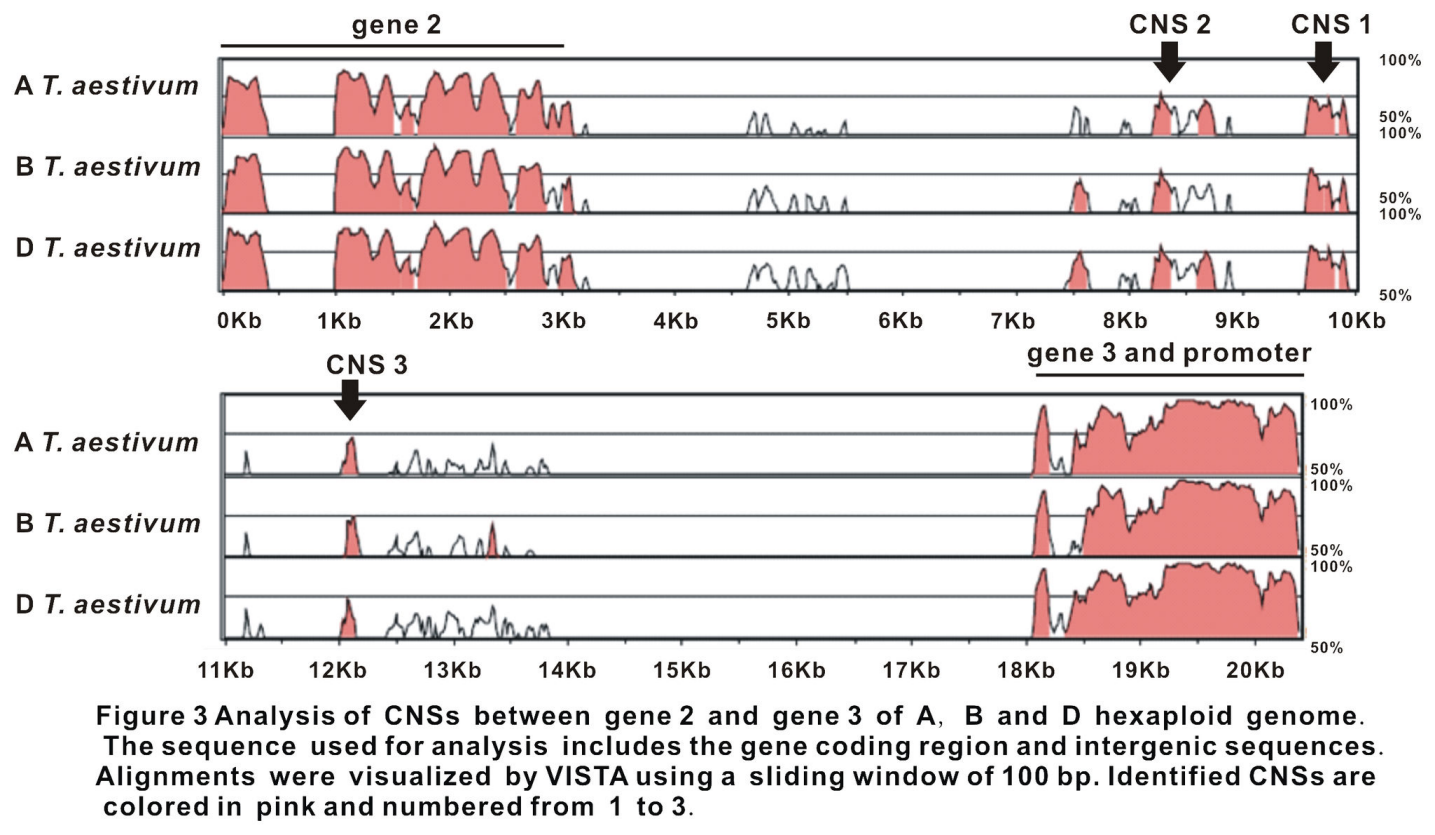
Analysis of CNSs between gene 2 and gene 3 of A, B and D hexaploid genome. The sequence used for analysis includes the gene coding region and intergenic sequences. Alignments were visualized by VISTA using a sliding window of 100 bp. Identified CNSs are colored in pink and numbered from 1 to 3.

Pair-wise comparisons of the orthologous *Rht-1* regions of the A, B, and D hexaploid were further performed to examine sequence divergence and conservation among the three wheat subgenomes. The CSR between the A (hexaploid) and B (hexaploid), A and D (hexaploid), B (hexaploid) and D (hexaploid), A (diploid) and B (tetraploid), B (tetraploid) and D (diploid), and A (diploid) and D (diploid) genome were 39.8%, 28.3%, 24.2%, 46.9%, 21.4% and 22.7%, respectively, and the CFS were 2,582bp, 2,066bp, 3,526bp, 1,739bp, 3,392bp, and 3,606bp. Both the CFS and CSR values were much smaller than those of homologous wheat genomes from different ploid levels (Table 2), suggesting that the more divergence among the three subgenomes as compared to the sequences of homologous genomes from different wheat ploids. In our comparative analysis, only six regions were found to be conserved across these three wheat subgenomes (Figure 2, S3). Of them, I, II and III regions contained genes 1, 2 and 3, respectively. The gene 1 was obviously lacking in the sequence of B genome likely due to the fact that the sequenced BAC region did not cover the sequence of the gene. Besides the above-mentioned gene regions, there were two sequence regions, Regions IV and V, containing three CNSs across all the three wheat genomes. The average length of CNS 1-3 is about 525 bp, 559 bp and 676 bp, respectively. CNS 1 and CNS 2 were located about 10kb and 8kb downstream region of gene 2 (D genome), respectively. CNS 3 was located about 6,000 bp upstream region of *Rht-1* (D genome) (Figure 3). These CNSs from different subgenomes had sequence similarities at least over 80% (Figure S4), and belong to unknown sequences.

Several other conserved sequence regions shared only by two genomes, but not by all three subgenomes were also detected. For instance, conserved region VI was observed to be shared by the A and D genomes (Figure 2A). Except for regions II, III, IV and V, none of other sequences was shared by the B and D genomes (Figure 2B). Region VII, a conserved region that was composed of unknown sequences, was only present between the A and B genomes (Figure 2C). We also compared the orthologous *Rht-1* regions between *T* *. urartu* and *Ae* *. tauchii* (Figure 2D). Regions VIII and IX contains CNS 1 and CNS 2, CNS 3, respectively, were shared by the two genomes, except for regions II and III. Comparison results between *T* *. urartu* and *B* *T* *. durum* showed that there have three conserved regions X (contains CNS 1 and CNS 2), XI (same with region VII) and XII (contains CNS 3), except for gene regions II and III (Figure 2E). Region XIII and XIV contains CNS 1 and CNS 2, CNS 3, respectively, were shared by B *T* *. durum* and D Ae*. tauchii* genomes (Figure 2F).

The nucleotide substitution rates (NSR) in the *Rht-1* and *DUF6*-like gene regions from the A, B, and D genomes of the hexaploid wheat were much higher as compared to the NSR between any two homologous wheat genomes from different ploid levels (Table 3; Table S9). Phylogenetic analysis has allowed us to establish evolutionary relationships (orthology *versus* paralogy) between the different members of the *Rht-1* and *DUF6*-like genes in wheat species and from different ploid levels. Although the two (AB) or three (ABD) subgenomes have been co-evolving in the tetraploid or hexaploid wheat species, phylogenetic inferences based on the *Rht-1* and *DUF6*-like gene sequences showed that both genes formed clusters that placed the gene sequences from the homologous genomes together with strong bootstrap supports (Figure S5).

Both *DUF6*-like and *Rht-1* appeared to be under strong purifying selection as evidenced by *Ka*/*Ks* values much less than 1 (Table 3, Table S9). Considerable variation in nucleotide substitutions was observed between these two genes, suggesting that they evolved at different rates; the number of nucleotide substitutions per site in the *Rht-1* gene CDS region is greater than that in the *DUF6*-like gene (*P*< 0.001) besides D: *Ae* *. tauschii* -D: *T. aestivum*, indicating that the former probably evolved faster than the latter (Table 4).

**Table 4:** The estimated divergence times between the wheat and related grasses based on pairwise comparisons of *DUF6*-like and *Rht-1* genes.

**Pairwise Genomes**	Substitution rates			Divergence	Substitution rates	Divergence	Average Divergence
	Coding-regions	Introns	Averages	Times (MYA)*	Coding-regions	Times (MYA)*	Times (MYA)*
	*DUF6-like*				***Rht***		
**A: *T* *. urartu* -A:T*.durum***	0.0068	0.0094	0.0081	0.2893	0.0329	2.2671	1.2782
**A: *T* *. urartu* -A:T*.aestivum***	0.0068	0.0094	0.0081	0.2893	0.0329	2.2671	1.2782
**A: *T* *. urartu* -B: *T* *. durum* **	0.0585	0.0094	0.0340	1.2124	0.3227	22.2373	11.7248
**A: *T* *. urartu* -D: *Ae* *. tauschii* **	0.0666	0.0613	0.0640	2.2837	0.2003	13.8027	8.0432
**A: *T* *. durum* -A:T*.aestivum***	0.0000	0.0000	0.0000	0.0000	0.0000	0.0000	0.0000
**B: *T* *. durum* -B: *T. aestivum***	0.0000	0.0000	0.0000	0.0000	0.0080	0.5513	0.2756
**B: *T* *. durum* -D: *Ae* *. tauschii* **	0.0418	0.0996	0.0707	2.5248	0.2558	17.6272	10.0760
**D: *Ae* *. tauschii* -D: *T. aestivum***	0.0031	0.0000	0.0016	0.0554	0.0000	0.0000	0.0277
**A: *T. aestivum*-B: *T. aestivum***	0.0658	0.1245	0.0952	3.3979	0.2949	20.3216	11.8597
**A: *T. aestivum*-D: *T. aestivum***	0.0705	0.0611	0.0658	2.3498	0.2409	16.6004	9.4751
**B: *T. aestivum*-D: *T. aestivum***	0.0384	0.0996	0.0690	2.4641	0.2487	17.1379	9.8010
**D: *T. aestivum-B.distachyon***	0.2832	0.7398	0.5115	18.2662	0.8594	59.2213	38.7438
**D: *T. aestivum-S.bicolor***	0.5683	1.8217	1.1950	42.6748	0.9806	67.5732	55.1240
**D: *T. aestivum-S.italica***	0.6111	1.3976	1.0044	35.8664	0.8741	60.2343	48.0504
**D: *T. aestivum-O.sativa***	0.4948	2.9005	1.6977	60.6249	1.0089	69.5234	65.0742
**D: *T. aestivum-Z.mays***	0.6321	1.6672	1.1497	41.0553	1.2046	83.0091	62.0322
***O. sativa- S. bicolor***	0.5414	2.8189	1.6802	60.0000	0.8707	60.0000	60.0000

* Note that 0.0000 in this table is the value after rounding, not the really zero.

### Sequence structure and molecular evolution of the orthologous *Rht-1* regions across different grass species

**Figure 4:**
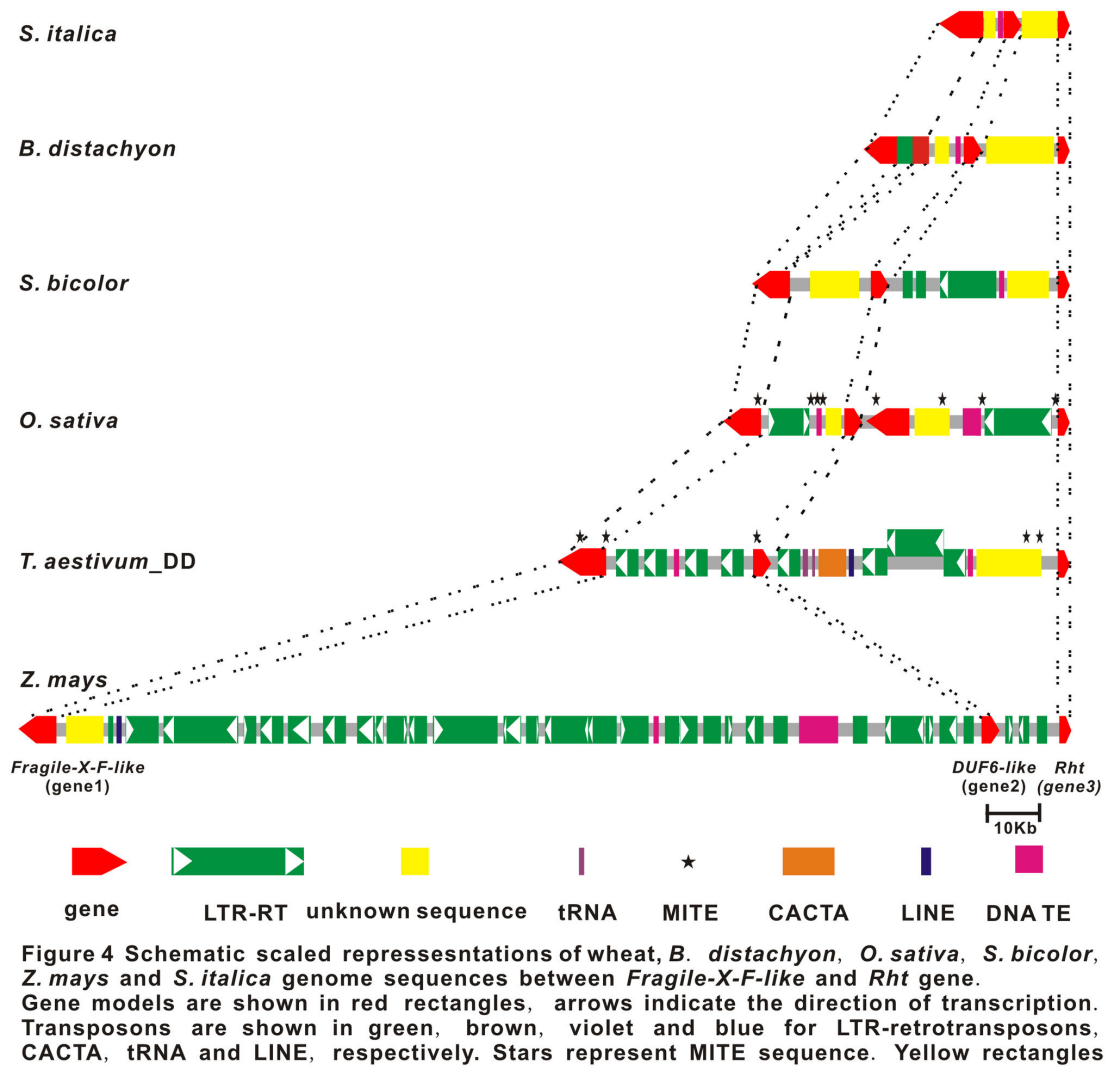
Schematic scaled repressesntations of wheat, *B* *. distachyon* , *O. sativa*, *S.* bicolor, *Z. mays* and *S* *. italica* genome sequences between *Fragile-X-F-like* and *Rht* gene. Gene models are shown in red rectangles, arrows indicate the direction of transcription. Transposons are shown in green, brown, violet and blue for LTR-retrotransposons, CACTA, tRNA and LINE, respectively. Stars represent MITE sequence. Yellow rectangles represent unknown sequences. Broken lines represent homologous region.

Comparisons of a 92,116-bp segment of the D subgenome in *T. aestivum* with orthologous genomic regions from *B* *. distachyon* (37,675-bp), *O. sativa* (65,935-bp), *S. bicolor* (58,435-bp), *Z. mays* (190,275-bp), and *S* *. italica* (24,234-bp) (Figure 4; Table S10) revealed a high synteny conservation of orthologous genes but large genomic divergence in the intergenic regions. The three genes, *Fragile-X-F*-like, *DUF6*-like and *Rht-1* in the wheat genomes, showed a highly conserved collinearity in the gene order and transcriptional orientation across the five grass genomes. The only violation of microsynteny is the presence of an additional gene between the *DUF6*-like and *Rht-1* gene in rice genome (Figure 4). In addition, high gene sequence conservation was also observed across grass species (Figure S6). The *Fragile-X-F-*like gene harbored fourteen exons in all species, and thirteen of which were identical in length across the species (Figure S1A). While all species had eight exons within the *DUF6*-like gene, seven of which were identical in length between all grasses (Figure S1B). Comparisons of amino acid sequences of the intronless *Rht-1* homologous genes suggested that these proteins contained conserved domains (N-terminal DELLA, TVHYNP motifs, C-terminal VHIID, LHR I, LHR II, PFYRE and SAW domain) and non-conserved domains including spacers between DELLA-TVHYNP and TVHYNP-Polys/T/V) (Figure S6C, S6D). The majority of the predicted amino acid sequences such as the C-terminal domain were highly conserved among these species with slightly variable lengths. Furthermore, we detected a CNS shared by all the surveyed grasses, which is in accordance with previous report [35].

We further determined phylogenetic relationships of *S* *. italica* , *B* *. distachyon* , *O. sativa*, *S.* bicolor, *Z. mays* and the wheat species based on amino acid sequences of both *DUF6*-like and *Rht-1* genes (Figure S5). Analyses of these two genes generated a similar topology with high bootstrap supports, which is fairly consistent to the commonly recognized evolutionary relationships of these grass species under study. The result suggested that *B* *. distachyon* is more closely related to wheat than the other four grass species, supporting the notion that *Brachypodium* can serve as a model plant for the analyses of the wheat genome [36].

The *Rht-1* homologous regions were shown to have various sizes in different grass genomes with the following order of *S* *. italica* < *B* *. distachyon* < *S. bicolor* < *O. sativa* < *T. aestivum* < *Z. mays* (Figure 4). Accordingly, gene density was the smallest in maize but largest in foxtail millet (Table S11). Further characterization of these *Rht-1* orthologous regions demonstrated that TEs have played an important role in determining the genome size variation, as indicated by 4.84%, 12.08%, 8.49%, 39.14%, 50.57% and 80.78% of transposable elements in foxtail millet, *Brachypodium* , sorghum, rice, wheat and maize, respectively (Table S11). Although we failed to detect any intact LTR retrotransposons in *B* *. distachyon* , sorghum and *S* *. italica* , a total of one, two and three intact retrotransposons were found in wheat, rice and maize, respectively (Table S12). The estimated insertion times of these retrotransposons ranged from 0 ~1.08 mya, indicating their active turnovers during the evolution of *Rht-1* genomic regions. Therefore, the expansion and contraction of the *Rht-1* homologous regions were mostly determined by the activities of retrotransposons.

### Divergence times of the wheat homologous genomes and related grasses

The divergence times between the major *Triticum* and *Aegilops* lineages of the wheat species and related grasses were separately estimated using intron and synonymous sites of the *Rht-1* and *DUF6*-like genes (Table 4). The results showed that the divergence of wheat with *B* *. distachyon* was more recent than that with other grass species but much earlier than that of the three wheat subgenomes. The diploid *Triticum* and *Aegilops* progenitors of the A, B and D genomes all radiated at approximately the same time, 9.4751-11.8597 MYA. The divergence times of the homologous A genomes are estimated to be 1.2782 MYA between the diploid and tetraploid and between diploid and hexaploid wheat, while the diploid D-genome species, *Ae* *. tauschii* , was found to have diverged from the hexaploid wheat only 0.0277 MYA (Table 4).

The identification of a few LTR retrotransposons that are intact and shared by two homologous genomes in the *Rht-1* regions permits us to further examine the sequence changes and divergence times of the homologous genomes in wheat. In this study, we used the colinear LTR retrotransposons identified in the *Rht-1* regions and dated their insertion time to estimate the divergence of the two homologous genomes (Table S4). Of a total of 13 intact colinear LTR retrotransposons, three shared by the A genomes, two by the B genomes, and one by the D genomes, were used to estimate the divergence times. Using the same molecular clock for dating the LTR retrotransposon insertions, we estimated the divergence time by calculating rates of nucleotide substitution between each pair of colinear retroelements to examine the variation in different sequences (Table 5). The estimated divergence time for the two homologous A genomes from the diploid and tetraploid and from diploid and hexaploid wheat is both around 0.68 million years ago (MYA). The divergence time of the two A genomes from the tetraploid and hexaploid wheat ranged from 0.00 to 0.03 MYA, with an average of 0.013 MYA. The two B genomes in tetraploid and hexaploid were estimated to have diverged in the last 0.01-0.03 MY, with an average of 0.02 MYA. The two D genomes from the diploid *Ae* *. tauschii* and hexaploid wheat diverged around 0.15 MYA (Table 5). It appeared that the approximations of the divergence times based on the colinear LTR retroelements were very similar with that using the sequence similarity of the above-mentioned two genes.

**Table 5:** Estimates of divergence time of the wheat genomes by comparisons of the shared LTR retrotransposons within the *Rht-1* homologous regions.

**LTR retrotransposons**		**Substitution rates**	**Divergence Times (MYA**)
**Shared in A Genome**			
*RLG_Sabrina_105A8-1* ^A^ : *RLG_Sabrina_1051O6-1* ^A^		0.0178	0.68
*RLG_Sabrina_105A8-1* ^A^ : *RLG_Sabrina_351D1-1* ^A^		0.0177	0.68
*RLG_Sabrina_1051O6-1* ^A^ : *RLG_Sabrina_351D1-1* ^A^		0.0001	0.00
*RLC_WIS_1051O6-1* ^A^ : *RLC_WIS_351D1-1* ^A^		0.0008	0.03
*RLC_WIS_1051O6-2* ^A^ : *RLC_WIS_351D1-2* ^A^		0.0002	0.01
**Shared in B Genome**			
*RLG_Fatima_315P18-1* ^B^ : *RLG_Fatima_17O6-1* ^B^		0.0003	0.01
*RLG_Fatima_315P18-2* ^B^ : *RLG_Fatima_17O6-2* ^B^		0.0009	0.03
**Shared in D Genome**			
*RLC_WIS_C4-3* ^D^ : *RLC_ WIS _1J9-7* ^D^		0.0038	0.15

## Discussion

### Repetitive sequences were the main elements that have a great influence on conservation

In the present study, several parameters including CSR, CFS, NSR and divergence time were employed to examine the conservation/divergence in the *Rht-1* regions among the three subgenomes, three ploids and the grass species. Our results revealed that the variation of conservation/divergence were dynamic when the sequences from the different ploidy levels, subgenomes, and grass genomes were compared. Repetitive sequences were the main elements that have a great influence on conservation. In present study, the main variation among the species and ploids were from the repetitive sequences, their insertion and/or deletion. The insertion time was around 2 MYA, much later than the divergence of the three subgenomes, suggesting that wheat genome expansion occurred after the subgenome divergence. Because of the rapid amplification and probably deletion of TEs, the intergenic regions among wheat subgenomes are largely divergent. The repetitive sequence not only produced the variation among the intergenic regions, but also in the genic regions. For example, a new TRIM transposon inserted in the DELLA domain of *Rht-B1*, and caused strongly reduced plant height [15,16].

Despite the large sequence divergence among subgenomes, we detected a high conservation between homologous wheat genomes, especially those A and B genomes from the tetraploid and hexaploid wheat and the D genomes from *Ae* *. tauschii* and hexaploid wheat. These results provide molecular supports to the current breeding practice that the tetraploid wheat and *Ae* *. tauschii* species are often used for modern wheat improvement.

### The inter subgenome CNSs in wheat

In our study, we identified three CNSs shared by the three wheat subgemones. CNSs are often rich in regulatory elements that may be involved in various biological functions. Uchida et al. reported that a K-box and RB-box in the CNSs upstream of *Knotted1* in grasses; *SHOOT MERISTEMLESS* (STM) in *Arabidopsis* regulate the gene expression of STM [37]. Another non-coding region which contains maize-sorghum-rice CNSs has been confirmed to serve as a *cis*-acting transcription-regulatory role [38]. The conservation of different CNSs could vary among plant species. The CNSs conserved among all the plant species, can be named as inter plant genome CNSs, while the CNSs conserved among the grass species can be regarded as inter grass genome CNSs [35,39]. The three CNSs reported in present study were conserved only among the three wheat subgenomes, so they were named as inter subgenome CNSs. We can speculate that widely distributed CNSs might serve more general biological functions, such as these by house-keeping genes. Meanwhile, CNSs shared by a small set of genomes might have a specific function. Hence, the inter subgenme CNSs may play an important role to retain the homoeologous relationship of three subgenomes in wheat. The inter subgenome CNSs also suggest that the three subgenomes share a common ancestor. With the fast progress of wheat genome sequencing, more subgenome CNSs will be discovered, and their structure characters and function will be discovered in the near future.

### Evolution of the *Rht-1* locus in different species

Comparative analyses of *Rht-1* locus regions from different species enhance our understanding of the structure and evolution of grass genomes. Divergence time analysis using the two gene sequences showed a significant overestimate of the time of tetraploid wheat formation, which occurred no more than 0.2756 Mya (Table 4). This upper estimate is fairly consistent with the previous reports [3,5]. The estimates based on synonymous substitutions are only approximate due to very low sequence divergence. Based on the nucleotide substitution rate of the two genes, the divergence times of A and B, A and D, and B and D were estimated to be 11.8597, 9.4751 and 9.8010 Mya, respectively. These estimated results are very consistent with the estimations based on the ACC1 loci [25]. The divergence of B genome might occur prior to the separation of A and D genomes. The diploid D genome was the latest genome added to the hexaploid wheat and showed high sequence conservation. The divergence time calculated based on *Rht* and *DUF6*-like gene together showed that *B* *. distachyon* and *O. sativa* diverged from each other only after they diverged from *Z. mays*. Several full-length LTR retroelements and two gene sequences were also employed to estimate the divergence times of *B* *. distachyon* and *T. aestivum* to be 38.7438 Mya, *S. bicolor* and *T. aestivum* to be 55.1240 Mya, *O. sativa* and *T. aestivum* to be 65.0742 Mya, *Z. mays* and *T. aestivum* to be 62.0322 Mya, and *S* *. italica* and *T. aestivum* to 48.0504Mya, respectively (Table 4). The *B* *. distachyon* - *T. aestivum* divergence time is within the range of a former estimate (35-40Mya) based on multiple gene sequences [40].

#### Accession code

DNA sequencing data are deposited in the National Center for Biotechnology Information (http://www.ncbi.nlm.nih.gov/) under accession number JX978696, KF282627, KF282628, KF282629, KF282630,HQ435325,HQ435330.

## Supporting Information

Figure S1 **Structure of Fragile-X-*F-like*, *DUF6*-like and *Rht* homologous genes between wheat subgenomes, B. *distachyon*, *O. sativa*, S. bicolor, *Z. mays* and *S* *. italica* , and expression pattern of the genes predicated in the *Rht-1* homologous regions.** (A) Structure of Fragile-X-F-like gene; (B), Structure of *DUF6*-like gene; (C), Structure of *Rht* gene; (D), 1, root, 2, stem, 3, leaf, 4, seed; Straight line boxes represent exons of identical size for the all species, bending line boxes represent exons of different size, while lines represent introns.
(TIF)

Figure S2 **Schematic scaled repressesntations of different polyploid levels of A, B, and D genomes, respectively.** Gene models are shown in red rectangles, arrows indicate the direction of transcription. rectangles. Transposons are shown in green, brown, violet and blue for LTR-retrotransposons, CACTA, tRNA and LINE, respectively. Stars represent MITE sequence. Yellow rectangles represent unknown sequences. (A) Different polyploid levels of A genomes; (B), Different polyploid levels of B genomes; (C), Different polyploid levels of D genomes; Broken lines represent homologous region.
(TIF)

Figure S3 **Compare of collinear regions between A, B and D genomes.** Gene models are shown in red rectangles; arrows indicate the direction of transcription. Transposons are shown in green, brown, violet and blue for LTR-retrotransposons, CACTA, tRNA and LINE, respectively. Stars represent MITE sequence. Yellow rectangles represent unknown sequences. Broken lines represent homologous region.
(TIF)

Figure S4 **Comparison of nucleotide sequence of CNS between different subgenomes.** The sequences of CNSs from the wheat subgenomes are used for alignment with ClustalX. Gray showed the same nucleotide bases between different subgenomes. (A), CNS1; (B), CNS2;(C),CNS3.
(TIF)

Figure S5 **Phylogenetic trees based on coding sequence data of *DUF6*-like and *Rht* genes.** Protein sequences of *DUF6*-like and *Rht* genes are used to construct the phylogenetic tree. (A), *DUF6*-like gene phylogenetic tree; (B), *Rht* gene phylogenetic tree. The trees were constructed by MEGA4.0 with neighbor-Joining (NJ) and bootstrap of replications 100.
(TIF)

Figure S6 **Comparison of structure and nucleotide sequence of gene1, gene2 and gene3 between different grasses.** The sequences of genes from the wheat and others grasses are used for alignment with ClustalX. Gray showed the same amino acid between different genomes. 1, A *T* *. urartu* ; 2, A *T* *. durum* ; 3, A *T. aestivum*; 4, B *T* *. durum* ; 5, B *T. aestivum*; 6, D Ae*. Tauschii*; 7, D *T. aestivum*; 8, *B* *. distachyon* ; 9, *O. sativa*; 10, *S. bicolor*; 11, *Z. mays*; 12, *S* *. italica* . (A), gene 1; (B), gene 2; (C) and (D), gene 3.
(TIF)

Table S1 **Information of predicted genes from the assembled BACs of the wheat genomes.**
(XLS)

Table S2 **Overall percentages of different TE classes identified within the wheat genomes.**
(DOC)

Table S3 **Intact LTR retrotransposons within the *Rht-1* homologous regions of the wheat genomes.**
(DOC)

Table S4 **Identification of the complete CACTA elements in the wheat genomes.**
(DOC)

Table S5 **Characterization of complete MITEs in the wheat genomes.**
(DOC)

Table S6 **The prediction of the wheat miRNAs and their target genes in the wheat genomes.**
(DOC)

Table S7 **Identification of microsatellites from the wheat A, B and D genomes and related grass genomes.**
(DOC)

Table S8 **The estimated evolutionary rates between the wheat and related grasses based on pairwise comparisons of *DUF6-like* gene.**
(DOC)

Table S9 **Sequence length variation of the *Rht-1* homologous regions of different grass genomes.**
(DOC)

Table S10 **Prediction of *cis*-acting regulatory elements within *Rht-1* homologous regions of the wheat genomes and related grass species.**
(DOC)

Table S11 **Overall percentages of genes, intergenic regions and TE classes identified in different grass genomes.**
(DOC)

Table S12 **Intact LTR retrotransposons within the *Rht-1* homologous regions of different related grass genomes.**
(DOC)
