# Supplementary figures and images for: Dynamic Evolution of Rht-1 Homologous Regions in Grass Genomes
Source: PLoS One. 2013 Sep 24;8(9):e75544. doi: 10.1371/journal.pone.0075544 (PMC3782514; doi:10.1371/journal.pone.0075544)

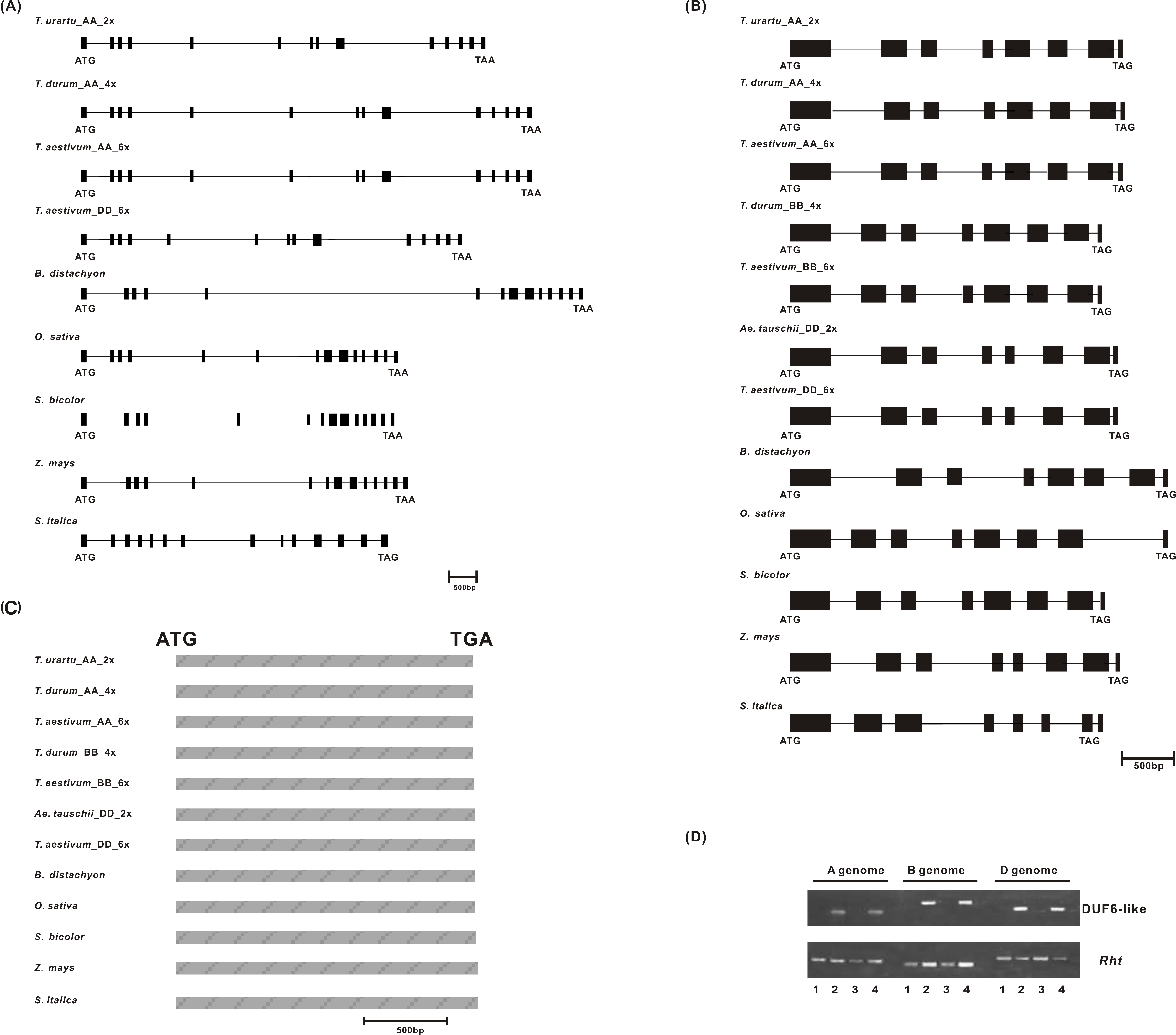

Supplement: Figure S1 — Structure of Fragile-X-F-like, DUF6-like and Rht homologous genes between wheat subgenomes, B. distachyon, O. sativa, S. bicolor, Z. mays and S . italica , and expression pattern of the genes predicated in the Rht-1 homologous regions. (A) Structure of Fragile-X-F-like gene; (B), Structure of DUF6-like gene; (C), Structure of Rht gene; (D), 1, root, 2, stem, 3, leaf, 4, seed; Straight line boxes represent exons of identical size for the all species, bending line boxes represent exons of different size, while lines represent introns. (TIF) [file pone.0075544.s001.tif]

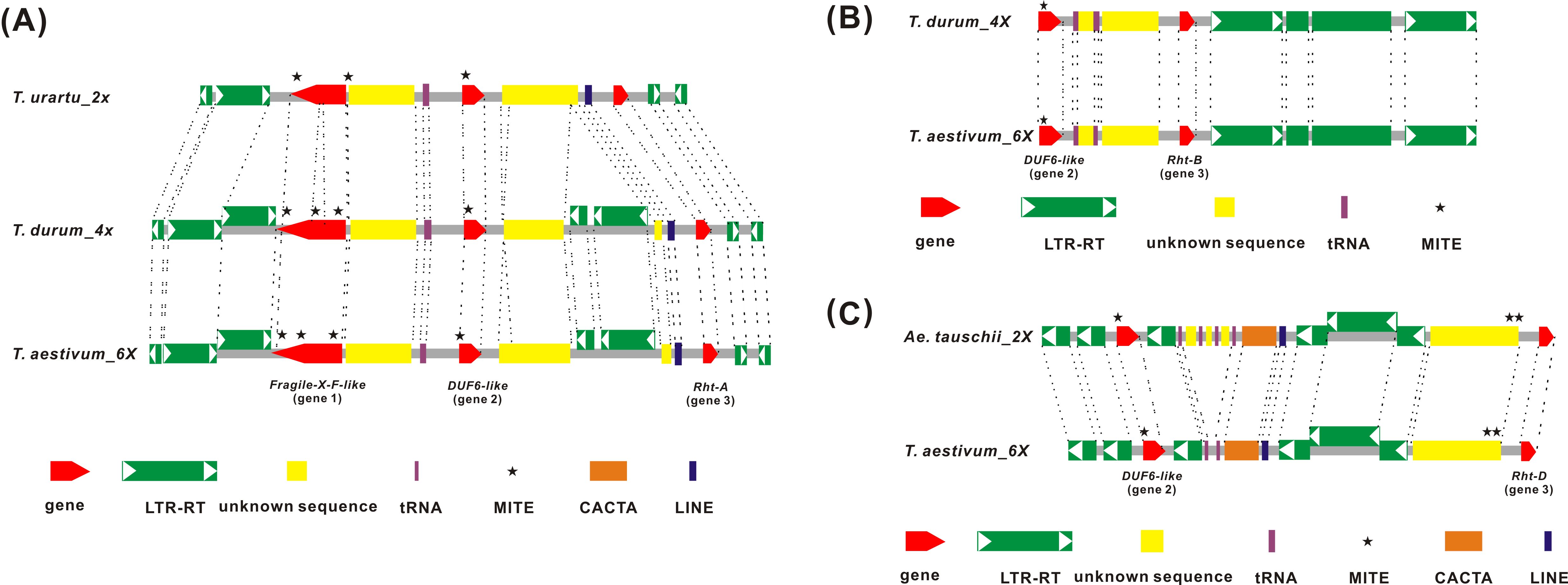

Supplement: Figure S2 — Schematic scaled repressesntations of different polyploid levels of A, B, and D genomes, respectively. Gene models are shown in red rectangles, arrows indicate the direction of transcription. rectangles. Transposons are shown in green, brown, violet and blue for LTR-retrotransposons, CACTA, tRNA and LINE, respectively. Stars represent MITE sequence. Yellow rectangles represent unknown sequences. (A) Different polyploid levels of A genomes; (B), Different polyploid levels of B genomes; (C), Different polyploid levels of D genomes; Broken lines represent homologous region. (TIF) [file pone.0075544.s002.tif]

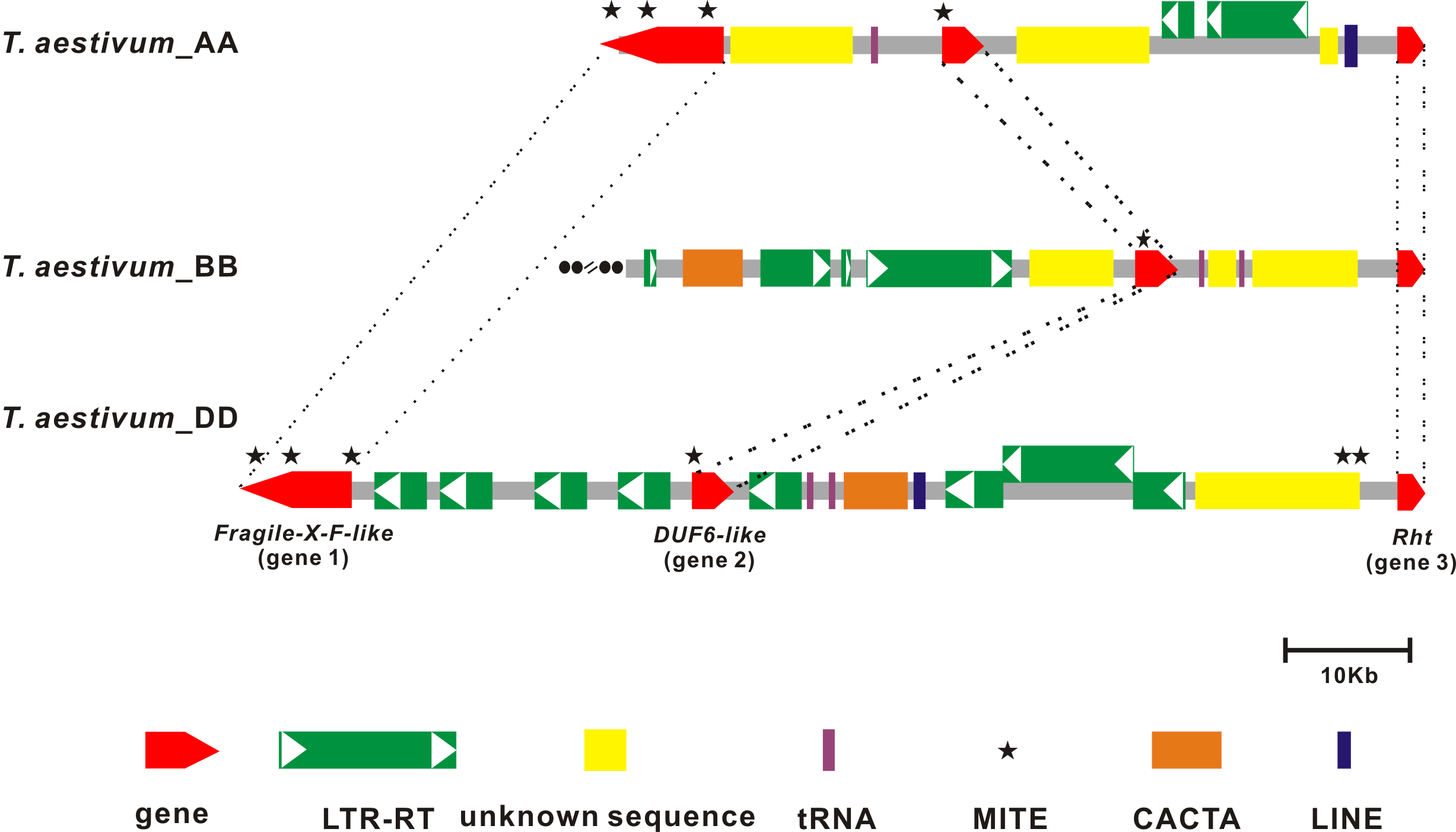

Supplement: Figure S3 — Compare of collinear regions between A, B and D genomes. Gene models are shown in red rectangles; arrows indicate the direction of transcription. Transposons are shown in green, brown, violet and blue for LTR-retrotransposons, CACTA, tRNA and LINE, respectively. Stars represent MITE sequence. Yellow rectangles represent unknown sequences. Broken lines represent homologous region. (TIF) [file pone.0075544.s003.tif]

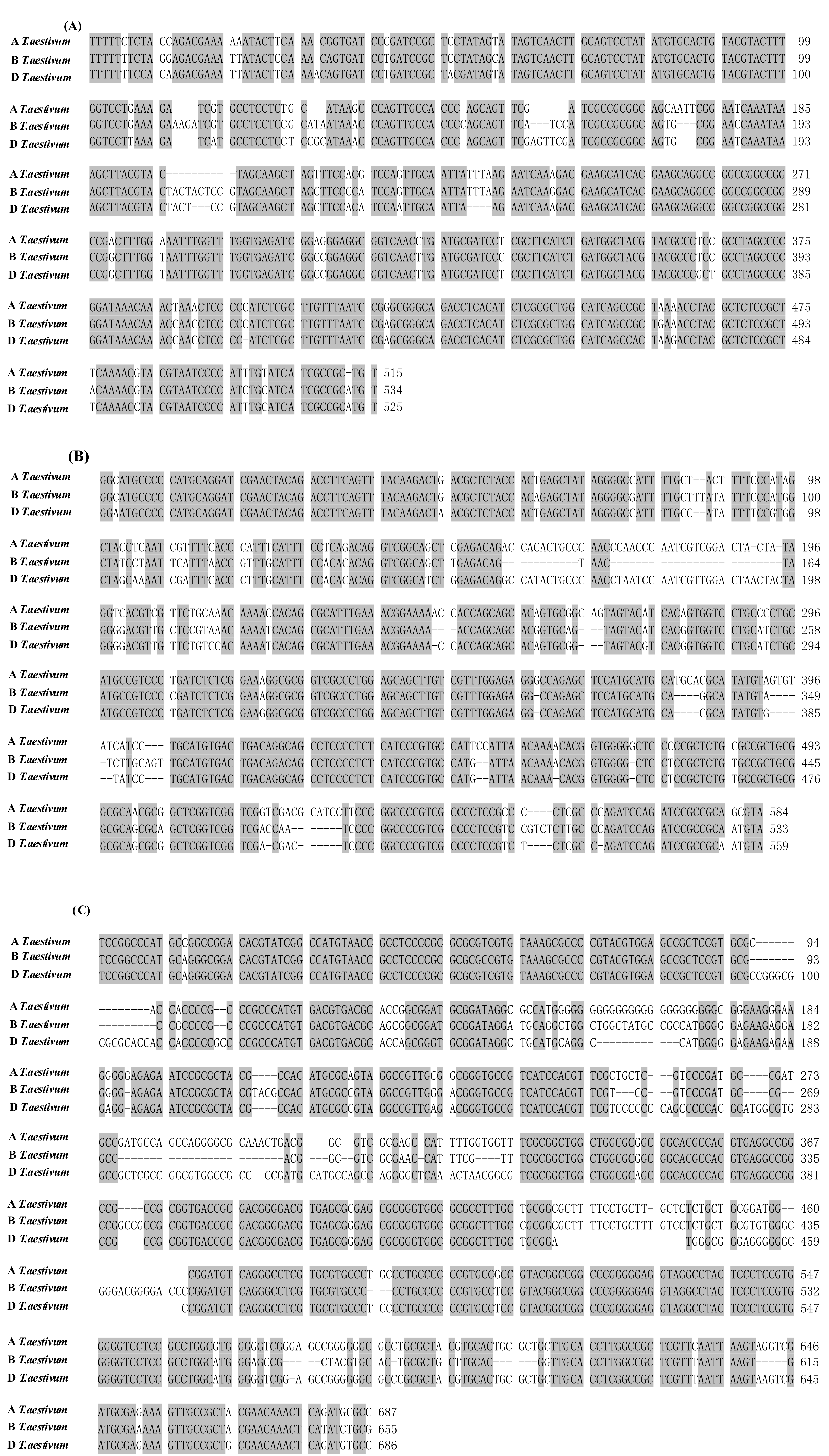

Supplement: Figure S4 — Comparison of nucleotide sequence of CNS between different subgenomes. The sequences of CNSs from the wheat subgenomes are used for alignment with ClustalX. Gray showed the same nucleotide bases between different subgenomes. (A), CNS1; (B), CNS2;(C),CNS3. (TIF) [file pone.0075544.s004.tif]

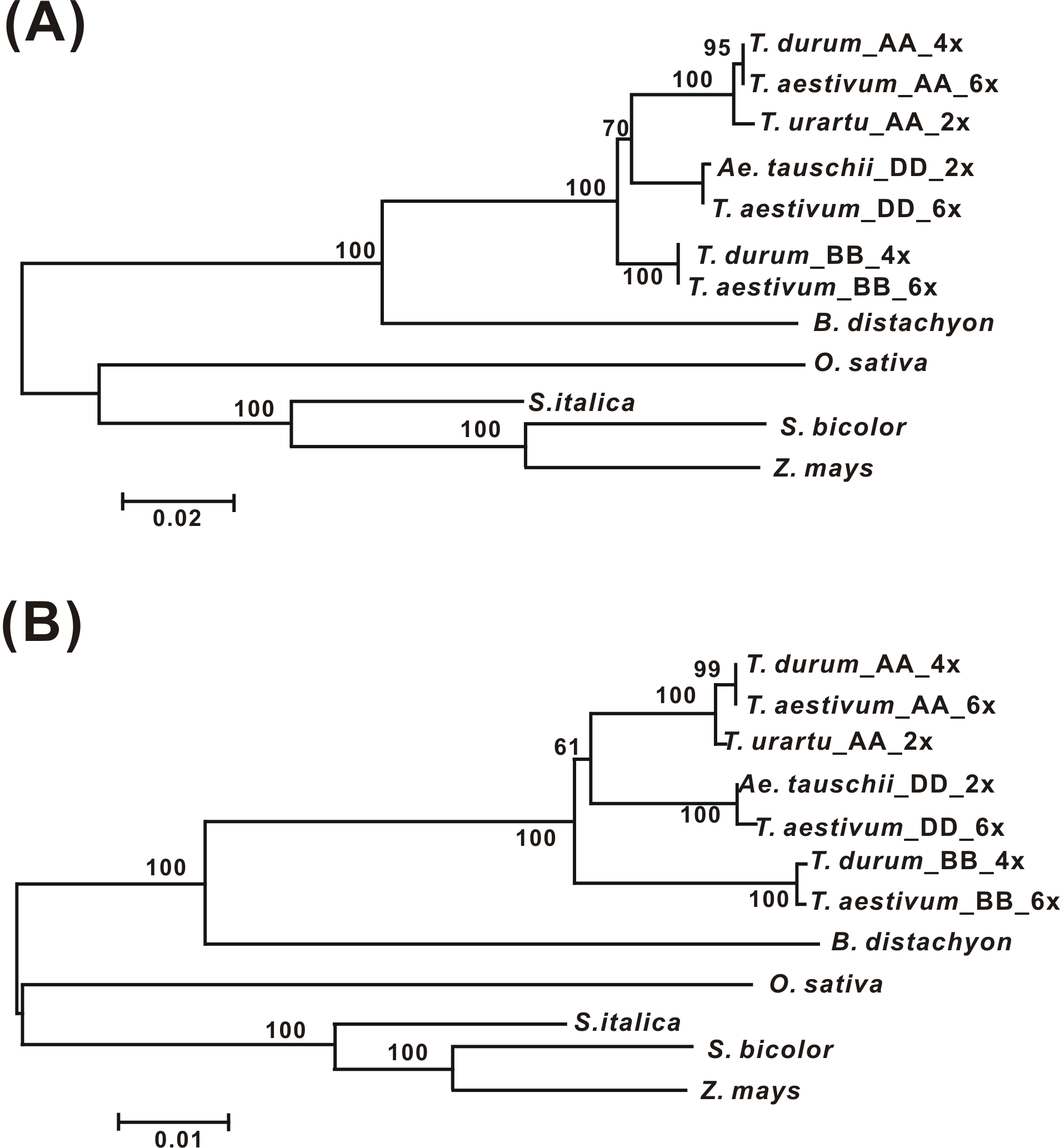

Supplement: Figure S5 — Phylogenetic trees based on coding sequence data of DUF6-like and Rht genes. Protein sequences of DUF6-like and Rht genes are used to construct the phylogenetic tree. (A), DUF6-like gene phylogenetic tree; (B), Rht gene phylogenetic tree. The trees were constructed by MEGA4.0 with neighbor-Joining (NJ) and bootstrap of replications 100. (TIF) [file pone.0075544.s005.tif]
