# Supplementary material for: Dynamic Evolution of Rht-1 Homologous Regions in Grass Genomes
Source: PLoS One. 2013 Sep 24;8(9):e75544. doi: 10.1371/journal.pone.0075544 (PMC3782514; doi:10.1371/journal.pone.0075544)
Supplement: Table S2 — Overall percentages of different TE classes identified within the wheat genomes. (DOC) [file pone.0075544.s008.doc]

**Table S2. Prediction of *cis*-acting regulatory elements within *Rht1* homologous regions of the wheat genomes and related grass species**

| **Sites** | **Functions** | **A genome** | **B genome** | **D genome** | **Brachypodium** | **Rice** | **Sorghum** | **Maize** | **Foxtail millet** |
| --- | --- | --- | --- | --- | --- | --- | --- | --- | --- |
| ***TATA***  ***CAAT***  ***GC-box***  ***A-box***  ***ABRE*** | determine the choice of gene transcription start  control of transcription initiation frequency  activate the transcription  cis-acting regulatory element | **√**  **√**  **√**  **√** | **√**  **√**  **√**  **√** | **√**  **√**  **√**  **√** | **√**  **√**  **√**  **√** | **√**  **√**  **√**  **×** | **√**  **√**  **√**  **√** | **√**  **√**  **√**  **√** | **√**  **√**  **√**  **√** |
|  | cis-acting element involved in the abscisic acid responsiveness | **√** | **√** | **√** | **√** | **√** | **×** | **√** | **√** |
| ***Sp1 motif*** | light responsive element | **√** | **√** | **√** | **√** | **√** | **√** | **√** | **√** |
| ***Skn-1 motif*** | cis-acting regulatory element required for endosperm expression | **√** | **√** | **√** | **√** | **√** | **×** | **×** | **√** |
| ***MBS*** | MYB binding site involved in drought-inducibility | **√** | **√** | **√** | **√** | **√** | **√** | **√** | **√** |
| ***G-box*** | cis-acting regulatory element involved in light responsiveness | **√** | **√** | **×** | **√** | **√** | **√** | **√** | **×** |
| ***P-box*** | gibberellin-responsive element | **×** | **×** | **√** | **×** | **×** | **×** | **×** | **×** |
| ***5’UTR Py-rich stretch*** | cis-acting element conferring high transcription levels | **×** | **√** | **√** | **√** | **√** | **×** | **×** | **√** |
| ***GCN4_motif*** | cis-regulatory element involved in endosperm expression | **√** | **√** | **√** | **×** | **×** | **√** | **√** | **×** |
| ***circadian*** | cis-acting regulatory element involved in circadian control | **√** | **√** | **×** | **×** | **√** | **×** | **×** | **√** |
| ***as-2-box*** | involved in shoot-specific expression and light responsiveness | **√** | **√** | **√** | **×** | **×** | **×** | **×** | **√** |
| ***TC-rich repeats*** | cis-acting element involved in defense and stress responsiveness | **√** | **√** | **√** | **×** | **√** | **√** | **√** | **√** |

√ represents the elements which were found in a genome, while × indicates that the element could not be detected.
