# Supplementary material for: Dynamic Evolution of Rht-1 Homologous Regions in Grass Genomes
Source: PLoS One. 2013 Sep 24;8(9):e75544. doi: 10.1371/journal.pone.0075544 (PMC3782514; doi:10.1371/journal.pone.0075544)
Supplement: Table S3 — Intact LTR retrotransposons within the Rht-1 homologous regions of the wheat genomes. (DOC) [file pone.0075544.s009.doc]

**Table S3. Overall percentages of different TE classes identified within the wheat genomes**

| **BACs** | **Retrotransposons** | | | | | **DNA transposons** | | | | | **Total** |
| --- | --- | --- | --- | --- | --- | --- | --- | --- | --- | --- | --- |
|  | **Ty1/copia** | **Ty3/gypsy** | **LINE** | **SINE** | **Total** | **CACTA** | **Mutator** | **Harbinger** | **Mariner** | **Total** |  |
| **A8** | 14.13 | 21.83 | 0.24 | 0.29 | 36.66 | - | - | 0.21 | 0.49 | 0.70 | 37.36 |
| **1051O6** | 26.21 | 20.75 | 0.29 | 0.17 | 47.42 | - | - | 0.16 | 0.47 | 0.63 | 48.05 |
| **351D1** | 23.24 | 12.75 | 0.35 | 0.22 | 36.56 | - | - | 0.33 | 0.60 | 0.93 | 37.49 |
| **315P18** | 13.53 | 22.01 | - | 0.09 | 35.63 | 3.13 | - | 0.28 | 0.47 | 3.88 | 39.51 |
| **17O6** | 12.67 | 39.10 | - | 0.13 | 51.90 | 4.17 | - | - | 0.32 | 4.49 | 56.39 |
| **C4** | 20.32 | 40.55 | 5.27 | - | 66.14 | 8.64 | 0.09 | 0.16 | 0.28 | 9.17 | 75.31 |
| **1J9** | 39.65 | 13.97 | 6.24 | - | 59.86 | 10.53 | 0.41 | - | 0.51 | 11.44 | 71.31 |
