# Supplementary material for: Dynamic Evolution of Rht-1 Homologous Regions in Grass Genomes
Source: PLoS One. 2013 Sep 24;8(9):e75544. doi: 10.1371/journal.pone.0075544 (PMC3782514; doi:10.1371/journal.pone.0075544)
Supplement: Table S4 — Identification of the complete CACTA elements in the wheat genomes. (DOC) [file pone.0075544.s010.doc]

**Table S4. Intact LTR retrotransposons within the *Rht1* homologous regions of the wheat genomes**

| **Genomes** | **Species** | **Elements** | **Superfamily** | **Length(bp)** | **5’LTR(bp)** | **3’LTR(bp)** | **TSDs** | **LTR divergence** | **Divergence times (MYA)** |
| --- | --- | --- | --- | --- | --- | --- | --- | --- | --- |
| **A** | ***T. urartu*** | *RLG_Sabrina_105A8-1* ^A^ | *Gypsy* | 8257 | 1578 | 1562 | TTGTG | 0.032 | 1.23 |
|  | ***T. durum*** | *RLG_Sabrina_1051O6-1*^A^ | *Gypsy* | 8242 | 1575 | 1563 | TTGTG | 0.042 | 1.62 |
|  |  | *RLC_WIS_1051O6-1* | *Copia* | 8851 | 1828 | 1721 | GAAAA | 0.020 | 0.77 |
|  |  | *RLC_WIS_1051O6-2* | *Copia* | 8741 | 1713 | 1715 | TACGT | 0.007 | 0.27 |
|  | ***T. aestivum*** | *RLG_Sabrina_351D1-1*^A^ | *Gypsy* | 8243 | 1575 | 1563 | TTGTG | 0.040 | 1.54 |
|  |  | *RLC_WIS_351D1-1* | *Copia* | 8751 | 1728 | 1721 | GAAAA | 0.020 | 0.77 |
|  |  | *RLC_WIS_351D1-2* | *Copia* | 8742 | 1713 | 1715 | TACGT | 0.007 | 0.27 |
| **Average divergence time** | |  |  |  |  |  |  |  | 1.45 |
|  | |  |  |  |  |  |  |  |  |
| **B** | ***T. durum*** | *RLG_Fatima_315P18-1*^B^ | *Gypsy* | 9160 | 470 | 470 | GTGAT | 0.056 | 2.15 |
|  |  | *RLG_Fatima_315P18-2*^B^ | *Gypsy* | 9098 | 473 | 473 | ACGAC | 0.022 | 0.85 |
|  | ***T. aestivum*** | *RLG_Fatima_17O6-1*^B^ | *Gypsy* | 9160 | 470 | 470 | GTGAT | 0.056 | 2.15 |
|  |  | *RLG_Fatima_17O6-2*^B^ | *Gypsy* | 9096 | 473 | 473 | ACGAC | 0.021 | 0.81 |
| **Average divergence time** | |  |  |  |  |  |  |  | 1.49 |
|  | |  |  |  |  |  |  |  |  |
| ***D*** | ***Ae.tauschii*** | *RLC_WIS_C4-3*^D^ | *Copia* | 8558 | 1696 | 1699 | CGCCC | 0.008 | 0.31 |
|  | ***T. aestivum*** | *RLC_ WIS _1J9-7*^D^ | *Copia* | 9796 | 1807 | 1788 | CGCCC | 0.008 | 0.31 |
| **Average divergence time** | |  |  |  |  |  |  |  | 0.31 |
