# Supplementary material for: Dynamic Evolution of Rht-1 Homologous Regions in Grass Genomes
Source: PLoS One. 2013 Sep 24;8(9):e75544. doi: 10.1371/journal.pone.0075544 (PMC3782514; doi:10.1371/journal.pone.0075544)
Supplement: Table S5 — Characterization of complete MITEs in the wheat genomes. (DOC) [file pone.0075544.s011.doc]

**Table S5. Identification of the complete CACTA elements in the wheat genomes**

| **Genome** | **Species** | **Elements** | **TIRs** | **TSDs** |
| --- | --- | --- | --- | --- |
| **D** | *Ae. tauschii* | DTC_Jorge-C4-1 | CAAAAAAAAGACACATCCGTG | ATG |
|  |  | DTC_TAT2-C4-1 | CTAGAAAAAGGCTTA | ATC |
|  | *T. aestivum* | DTC_Vincent_1J9-1 | CAAGAAATATGTCAACTAGTGACC | TAA |
|  |  | DTC_Jorge-1J9-1 | CAAAAAAAGACACATCCGTG | TAG |
|  |  | DTC_TAT2-1J9-1 | CTAGAAAAAGGCTTA | ATC |
