# Supplementary material for: Dynamic Evolution of Rht-1 Homologous Regions in Grass Genomes
Source: PLoS One. 2013 Sep 24;8(9):e75544. doi: 10.1371/journal.pone.0075544 (PMC3782514; doi:10.1371/journal.pone.0075544)
Supplement: Table S6 — The prediction of the wheat miRNAs and their target genes in the wheat genomes. (DOC) [file pone.0075544.s012.doc]

| **Genomes** | **Species** | **Elements** | **Blast Results** | **Superfamily** | **Total**  **Length (bp)** | **TSD** |
| --- | --- | --- | --- | --- | --- | --- |
| **A** | *T. urartu* | DTT_Thalos_105A8-1 | TREP3107 | Stowaway | 165 | TA |
|  |  | DTT_Hades_105A8-1 | TREP222 | Stowaway | 104 | TA |
|  |  | MITE-105A8-3 |  | Stowaway | 94 | TA |
|  | *T. durum* | DTT_Thalos_1051O6-1 | TREP3107 | Stowaway | 165 | TA |
|  |  | MITE-1051O6-2 |  | Stowaway | 127 | TA |
|  |  | DTT_Hades_1051O6-1 | TREP222 | Stowaway | 104 | TA |
|  |  | MITE-1051O6-4 |  | Stowaway | 94 | TA |
|  | *T. aestivum* | DTT_Thalos_351D1-1 | TREP3107 | Stowaway | 165 | TA |
|  |  | MITE-351D1-2 |  | Stowaway | 127 | TA |
|  |  | DTT_Hades_351D1-1 | TREP222 | Stowaway | 104 | TA |
|  |  | MITE-351D1-4 |  | Stowaway | 94 | TA |
| **B** | *T. durum* | DTT_Hades_315P18-1 | TREP220 | Stowaway | 103 | TA |
|  | *T. aestivum* | DTT_Hades_17O6-1 | TREP220 | Stowaway | 103 | TA |
| **D** | *Ae. tauschii* | DTT_Thalos_C4-1 | TREP3107 | Stowaway | 163 | TA |
|  |  | DTT_Athos_C4-1 | TREP3081 | Stowaway | 85 | TA |
|  |  | DTT­_Thalos_C4-2 |  | Stowaway | 112 | TA |
|  | *T. aestivum* | DTT_Thalos_1J9-1 | TREP3107 | Stowaway | 163 | TA |
|  |  | DTT_Athos_1J9-2 | TREP3081 | Stowaway | 85 | TA |
|  |  | DTT­_Thalos_1J9-2 |  | Stowaway | 112 | TA |
|  |  | DTT_Icarus_1J9-1 | TREP3092 | Stowaway | 107 | TA |
|  |  | DTT_Hades_1J9-1 | TREP220 | Stowaway | 104 | TA |
|  |  | DTT_ Icarus_1J9-2 |  | Stowaway | 122 | TA |

**Table S6. Characterization of complete MITEs in the wheat genomes**
