# Supplementary material for: Dynamic Evolution of Rht-1 Homologous Regions in Grass Genomes
Source: PLoS One. 2013 Sep 24;8(9):e75544. doi: 10.1371/journal.pone.0075544 (PMC3782514; doi:10.1371/journal.pone.0075544)
Supplement: Table S7 — Identification of microsatellites from the wheat A, B and D genomes and related grass genomes. (DOC) [file pone.0075544.s013.doc]

**Table S7. The prediction of the wheat miRNAs and their target genes in the wheat genomes**

| **BACs** | **MiRNA families** | **Sequences** | **Predicted target**  **unigenes /ESTs** | **Target gene families** |
| --- | --- | --- | --- | --- |
| **105A8** | TamiR1122 | uagauacauccguaucuuaga | CJ564885 | unknown protein |
| **1051O6** | TamiR1137 | uaguacaaaguugagucauuc | BG905880/ Ta.12786 | unknown protein |
| **315P18** | TamiR1132 | auuauggaacggaaggag | CJ954495 | glutathione-S-transferase |
| **1J9** | TamiR1121 | aguaguuaucuaaacgcucuua | BJ282606/ Ta.34673 | ubiquitin-protein ligase |
