# Supplementary material for: Dynamic Evolution of Rht-1 Homologous Regions in Grass Genomes
Source: PLoS One. 2013 Sep 24;8(9):e75544. doi: 10.1371/journal.pone.0075544 (PMC3782514; doi:10.1371/journal.pone.0075544)
Supplement: Table S8 — The estimated evolutionary rates between the wheat and related grasses based on pairwise comparisons of DUF6-like gene. (DOC) [file pone.0075544.s014.doc]

**Table S8. Identification of microsatellites from the wheat A, B and D genomes and related grass genomes**

| **Genomes** | **A** | | | **B** | | | | **D** | | | | | | | | | | |
| --- | --- | --- | --- | --- | --- | --- | --- | --- | --- | --- | --- | --- | --- | --- | --- | --- | --- | --- |
| **BACs** | **105A8** | **1051O6** | **351D1** | **315P18** | **17O6** | | | **C4** | | | | **1J9** | | | | | | |
| **Motifs** | / | cgct | cgct | cta | ta | ta | cta | ct | aga | ga(ag) | tcgg | ga | aga | ga | tcgg | ct | ta | gtt |
| **No.**  **of repeats** | / | 5 | 5 | 11 | 38 | 34 | 11 | 23 | 9/6 | 9 | 9 | 15 | 14 | 11 | 7 | 15 | 26 | 7 |

| **Genomes** | ***S. italic*** | | ***B. distachyon*** | | ***S. bicolor*** | | | | ***O. sativa*** | | | | | ***Z. mays*** | | | | |
| --- | --- | --- | --- | --- | --- | --- | --- | --- | --- | --- | --- | --- | --- | --- | --- | --- | --- | --- |
| **Motifs** | ct | cct | ga | ct | ta | cga | cta | cct | | ct | ta | ta | gcctg | | tta | tc | ta | ag |
| **No.**  **of repeats** | 13 | 6 | 12 | 7 | 39 | 6 | 6 | 6 | | 11 | 11 | 37 | 5 | | 7 | 9 | 33 | 9 |
