# Supplementary material for: Dynamic Evolution of Rht-1 Homologous Regions in Grass Genomes
Source: PLoS One. 2013 Sep 24;8(9):e75544. doi: 10.1371/journal.pone.0075544 (PMC3782514; doi:10.1371/journal.pone.0075544)
Supplement: Table S9 — Sequence length variation of the Rht-1 homologous regions of different grass genomes. (DOC) [file pone.0075544.s015.doc]

**Table S9. The estimated evolutionary rates between the wheat and related grasses based on pairwise comparisons of *DUF6-like* gene**

| **Pairwise Genomes** | **5’UTR regions** | | | **Coding-regions** | | | **Introns 3’UTR regions** | | |
| --- | --- | --- | --- | --- | --- | --- | --- | --- | --- |
|  | **Length (bp)** | ***K*** | **Length**  **(bp)** | ***Ks*** | ***Ka*** | ***Ka*/*Ks*** | **Length**  **(bp)** | ***K*** | **Length *K***  **(bp)** |

| **A: *T. urartu-*A: *T. durum*** | 812 | 0.0104 | 1320 | 0.0068 | 0.0000 | 0.0010 | 1254 | 0.0094 | 355 | 0.0144 |
| --- | --- | --- | --- | --- | --- | --- | --- | --- | --- | --- |
| **A: *T. urartu-*A :*T. aestivum*** | 812 | 0.0104 | 1320 | 0.0068 | 0.0000 | 0.0010 | 1254 | 0.0094 | 355 | 0.0144 |
| **A: *T. urartu*-B: *T. durum*** | 819 | 0.0104 | 1323 | 0.0585 | 0.0000 | 0.0010 | 1257 | 0.0094 | 369 | 0.2050 |
| **A: *T. urartu*-D: *Ae.tauschii*** | 818 | 0.0697 | 1323 | 0.0666 | 0.0141 | 0.2117 | 1393 | 0.0613 | 396 | 0.1035 |
| **A: *T. durum-*A: *T. aestivum*** | 796 | 0.0000 | 1320 | 0.0000 | 0.0000 | 0.0010 | 1252 | 0.0000 | 355 | 0.0000 |
| **B: *T. durum-*B: *T. aestivum*** | 666 | 0.0000 | 1308 | 0.0000 | 0.0000 | 0.0010 | 1071 | 0.0000 | 349 | 0.0000 |
| **B: *T. durum* - D: *Ae. tauschii*** | 745 | 0.0878 | 1323 | 0.0418 | 0.0187 | 0.4474 | 1381 | 0.0996 | 397 | 0.0841 |
| **D: *Ae. tauschii*-D: *T. aestivum*** | 742 | 0.0052 | 1185 | 0.0031 | 0.0000 | 0.0010 | 1375 | 0.0000 | 395 | 0.0000 |
| **A: *T. aestivum-*B: *T. aestivum*** | 819 | 0.1085 | 1320 | 0.0658 | 0.0175 | 0.2660 | 1256 | 0.1245 | 369 | 0.2028 |
| **A: *T. aestivum-*D: *T. aestivum*** | 818 | 0.0705 | 1320 | 0.0705 | 0.0140 | 0.1986 | 1394 | 0.0611 | 396 | 0.1026 |
| **B: *T. aestivum-*D: *T. aestivum*** | 745 | 0.0791 | 1320 | 0.0384 | 0.0186 | 0.4844 | 1379 | 0.0996 | 397 | 0.0841 |
| **D: *T. aestivum-B. distachyon*** | 798 | 2.3346 | 1224 | 0.2832 | 0.0933 | 0.3295 | 1620 | 0.7398 | 403 | 0.5579 |
| **D: *T. aestivum-S. bicolor*** | 872 | 2.4428 | 1326 | 0.5683 | 0.1675 | 0.2947 | 1270 | 1.8217 | 408 | 2.5077 |
| **D: *T. aestivum-S. italica*** | 855 | 5.8223 | 1422 | 0.6111 | 0.1404 | 0.2298 | 1420 | 1.3976 | 551 | 2.7202 |
| **D: *T. aestivum-O. sativa*** | 787 | 1.2964 | 1362 | 0.4948 | 0.1355 | 0.2738 | 1641 | 2.9005 | 410 | 3.7342 |
| **D: *T. aestivum-Z. mays*** | 784 | 2.2463 | 1347 | 0.6321 | 0.1601 | 0.2533 | 1389 | 1.6672 | 407 | 8.7059 |
