# Supplementary material for: Dynamic Evolution of Rht-1 Homologous Regions in Grass Genomes
Source: PLoS One. 2013 Sep 24;8(9):e75544. doi: 10.1371/journal.pone.0075544 (PMC3782514; doi:10.1371/journal.pone.0075544)
Supplement: Table S10 — Prediction of cis-acting regulatory elements within Rht-1 homologous regions of the wheat genomes and related grass species. (DOC) [file pone.0075544.s016.doc]

**Table S10. Sequence length variation of the *Rht1* homologous regions of different grass genomes**

| **Species** | **Total length**  **(bp)** | **Intergenic regions**  **(bp)** | **Genic regions**  **(bp)** | ***Fragile-X-F-*like:*DUF6*-like**  **(bp)** | ***DUF6*-like:*Rht***  **(bp)** |
| --- | --- | --- | --- | --- | --- |
| ***S. italica*** | 24,234 | 14,834 | 10,400 | 3,125 | 11,709 |
| ***B. distachyon*** | 37,675 | 17,634 | 20,041 | 3,511 | 14,123 |
| ***S. bicolor*** | 58,435 | 46,363 | 12,072 | 14,324 | 32,039 |
| ***O. sativa*** | 65,935 | 53,476 | 12,459 | 14,423 | 39,053 |
| ***T. aestivum* (DD)** | 92,116 | 79,373 | 12,743 | 25,689 | 53,684 |
| ***Z. mays*** | 190,275 | 178,297 | 11,978 | 165,461 | 12,836 |
