# Supplementary material for: Dynamic Evolution of Rht-1 Homologous Regions in Grass Genomes
Source: PLoS One. 2013 Sep 24;8(9):e75544. doi: 10.1371/journal.pone.0075544 (PMC3782514; doi:10.1371/journal.pone.0075544)
Supplement: Table S11 — Overall percentages of genes, intergenic regions and TE classes identified in different grass genomes. (DOC) [file pone.0075544.s017.doc]

**Table S11. Overall percentages of genes, intergenic regions and TE classes identified in different grass genomes**

| **Genes, intergenic regions and**  **TE classes** | **% of**  **Brachypodium genomic region** | **% of**  **Sorghum genomic region** | **% of**  **Rice**  **genomic region** | **% of**  **Wheat**  **genomic region** | **% of**  **Maize**  **genomic region** | **% of**  **Foxtail Millet genomic region** |
| --- | --- | --- | --- | --- | --- | --- |
| **Genes** | **53.51** | **20.65** | **18.76** | **13.83** | **6.30** | **42.91** |
| **Intergenic regions** | **46.49** | **79.35** | **81.24** | **86.17** | **93.70** | **57.09** |
| **TEs** | **12.08** | **8.49** | **39.14** | **50.57** | **80.78** | **4.84** |
| **Class I elements (retrotransposons)** | **11.22** | **6.30** | **27.86** | **44.15** | **72.52** | / |
| **LTR retrotransposons** | 11.22 | 6.12 | 27.56 | 40.62 | 71.60 | / |
| ***gypsy*-like** | 0.72 | 6.21 | / | 24.57 | 36.88 | / |
| ***copia*-like** | 10.51 | 0.09 | 27.56 | 16.05 | 34.72 | / |
| **Non-LTR retrotransposons** | / | / | 0.30 | 3.53 | 0.92 | / |
| **LINEs** | / | / | 0.30 | 3.34 | 0.92 | / |
| **SINEs** | / | / | / | 0.19 | / | / |
| **Class II elements (DNA transposons)** | **0.68** | **1.62** | **9.74** | **6.42** | **8.09** | **3.07** |
| **CACTA** | / | / | / | 4.89 | / | / |
| **TcMar-Stowaway** | / | / | 1.47 | 0.56 | / | / |
| **MULE-MuDR/hAT/**  **Harbinger/CMC-EnSpm** | 0.68 | 1.62 | 8.27 | 0.97 | 8.09 | 3.07 |
| **Other known repeats** | 0.18 | 0.57 | 1.54 | / | 0.17 | 0.77 |
| **Simple repeats** | 0.30 | 0.36 | 0.34 | 1.14 | 0.10 | 0.81 |
| **Low complexity** | 0.20 | 0.74 | 0.81 | 1.10 | 0.14 | 0.78 |
