# Supplementary material for: Dynamic Evolution of Rht-1 Homologous Regions in Grass Genomes
Source: PLoS One. 2013 Sep 24;8(9):e75544. doi: 10.1371/journal.pone.0075544 (PMC3782514; doi:10.1371/journal.pone.0075544)
Supplement: Table S12 — Intact LTR retrotransposons within the Rht-1 homologous regions of different related grass genomes. (DOC) [file pone.0075544.s018.doc]

**Table S12. Intact LTR retrotransposons within the *Rht1* homologous regions of different related grass genomes**

| **Genomes** | **Superfamily** | **Length**  **(bp)** | **5’ LTR**  **(bp)** | **3’ LTR**  **(bp)** | **TSDs** | **Divergence times (MYA)** |
| --- | --- | --- | --- | --- | --- | --- |
| ***T. aestivum* (DD)** | *Copia* | 9796 | 1807 | 1788 | CGCCC | 0.31 |
| ***O. sativa*** | *Copia* | 11,934 | 1574 | 1549 | CCT(T)TA | 0.96 |
|  | *Copia* | 6318 | 915 | 916 | GACTA | 1.08 |
| ***Z. mays*** | *Gypsy* | 7407 | 671 | 671 | GTACG | 0.00 |
|  | *Gypsy* | 14040 | 1674 | 1676 | ACTAG | 1.08 |
|  | *Copia* | 8199 | 1246 | 1258 | ACGGG | 0.27 |
| ***B. distachyon**** | / | / | / | / | / | **/** |
| ***S. bicolor**** | / | / | / | / | / | **/** |
| ***S. italica**** | / | / | / | / | / | / |

***** No intact LTR retrotransposons were detected in *S. italic*, *B. distachyon* and *S. bicolor*.
